# Trends in Infectious Disease Mortality Rates, Spain, 1980–2011

**DOI:** 10.3201/eid2005.131528

**Published:** 2014-05

**Authors:** Teresa López-Cuadrado, Alicia Llácer, Rocio Palmera-Suárez, Diana Gómez-Barroso, Camelia Savulescu, Paloma González-Yuste, Rafael Fernández-Cuenca

**Affiliations:** Carlos III Institute of Health, Madrid, Spain (T. López-Cuadrado, A. Llácer, R. Palmera-Suárez, D. Gómez-Barroso, C. Savulescu, P. González-Yuste, R. Fernández-Cuenca);; Centros de Investigación Biomédica en Red de Epidemiología y Salud Pública, Madrid (A. Llácer, R. Palmera-Suárez, D. Gómez-Barroso, R. Fernández-Cuenca);; Complejo Hospitalario de Toledo, Toledo, Spain (P. Gónzalez-Yuste)

**Keywords:** mortality rates, infectious diseases, trends, international classification of diseases, international classification of diseases, ICD, joinpoints, Spain

## Abstract

Using mortality data from National Institute of Statistics in Spain, we analyzed trends of infectious disease mortality rates in Spain during 1980–2011 to provide information on surveillance and control of infectious diseases. During the study period, 628,673 infectious disease–related deaths occurred, the annual change in the mortality rate was −1.6%, and the average infectious disease mortality rate was 48.5 deaths/100,000 population. Although the beginning of HIV/AIDS epidemic led to an increased mortality rate, a decreased rate was observed by the end of the twentieth century. By codes from the International Classification of Diseases, 9th revision, the most frequent underlying cause of death was pneumonia. Emergence and reemergence of infectious diseases continue to be public health problems despite reduced mortality rates produced by various interventions. Therefore, surveillance and control systems should be reinforced with a goal of providing reliable data for useful decision making.

## Abstract 2

Surveillance and control systems should be reinforced to provide reliable data.

Although infectious diseases continue to account for considerable illness and death worldwide (*1**,**2*), mortality rates for these diseases in industrialized countries decreased considerably by end of the twentieth century (*1**–**4*). The decrease in infectious disease mortality rates was caused by a set of complex factors fundamentally linked to development, such as better sanitation as populations became more urban, and improvements in infrastructure, nutrition, and biotechnological advances, particularly in the field of vaccines and antimicrobial drugs (*5*). This reduction was reflected mainly in the decrease of child mortality rates. At the beginning of the twentieth century, 30% of all deaths caused by infectious diseases were among children <5 years of age; by the end of the twentieth century, these diseases accounted for only 1.4% of all deaths (*3*). In the 1980s, the decreasing trend of the infectious disease mortality rate in industrialized countries was interrupted by the HIV/AIDS epidemic, which confronted the scientific community and health authorities with the challenge of a new emerging infection that has still not been controlled.

In Spain, as in other industrialized countries, mortality rates decreased overall and for children over the course of the twentieth century, life expectancy increased dramatically. The progressive decrease in deaths from infectious causes was interrupted by the HIV/AIDS epidemic, which changed the trend and pattern of infectious diseases for the population overall and for specific age groups affected (*6*). New antiretroviral therapies introduced in the mid-1990s decreased deaths caused by HIV/AIDS, as well as deaths caused by other infectious diseases. However, other threats to human health related to emergence and reemergence of infectious disease have arisen (*7*), mainly because of environmental and climate changes, travel and trade, human behavior, new technologies, microbial adaptation, and host-impaired immunity (*8*). These continuous threats make specific infectious disease surveillance and control programs even more necessary (*8*).

All-cause and cause-specific mortality rates, as well as standardized mortality rates, are still good indicators for ascertaining the public health effects of a given disease and assessing trends in incidence. Successive revisions of the International Classification of Diseases (ICD) have continued to apply an etiologic criterion to pool part of infectious and parasitic diseases in a single group and leaving conditions of infectious origin in other groups. Pinner et al. (*9*) found that ICD codes for infectious and parasitic diseases in the ICD, 9th revision (ICD-9) included only 67% of the 1,131 codes that could be included as infectious diseases or consequences of infections, and applied comprehensive criteria to the analysis of infectious disease mortality rates. In Spain, infectious disease mortality rates in the early 1990s were assessed by using similar criteria and resulted in a 3-fold increase in number of deaths related to ICD codes for infectious and parasitic diseases (*6**,**10*). The purposes of the current study were to determine the magnitude of infectious disease mortality rates rate overall and by sex, age, and the principal causes implicated, and to describe trends during 1980–2011 to clarify surveillance needs and enhance control strategies.

## Methods

In Spain, the source of mortality rate statistics is the medical death certificate, a compulsory administrative document that is completed by the physician who certifies the death. Data are subsequently forwarded to the regional mortality registries where causes of death are coded according to ICD guidelines. According to World Health Organization recommendations, the cause of death that is ICD coded should be taken as the underlying cause of death (*11*).

We analyzed ICD codes of underlying causes of death provided by the Spanish National Statistics Institute (NSI). We selected deaths caused by infectious causes corresponding to ICD-9 codes for 1980–1998 and ICD-10 codes for 1999–2011. From the ICD-9 and ICD-10 codes, we selected all codes of infectious and parasitic diseases and other infectious causes from remaining groupings (Table 1). Of these diseases, 10 that accounted for >90% of all deaths (pneumonia, septicemia, cardiac infections, AIDS and HIV infection, renal infections, tuberculosis, acute respiratory infection, influenza, viral hepatitis, and intestinal infections) were independently selected. For HIV/AIDS, data collection began in 1989 when a newly created specific ICD-9 code was assigned and began to be used in Spain; related diseases were previously allocated to unspecified immunity disorders.

**Table 1:** Infectious diseases analyzed, Spain, 1980–2011*

Diseases/infections	ICD-9 codes	ICD-10 codes	No. cases		SR, 2007–2011	APC, % , 1980–2011
			1980	2011 (%)		
All causes			288,426	386,017		
Infectious diseases	NA	NA	19,106	22,646 (100)	1.5	−1.6†
Pneumonia	480–486	J12–J18	9,292	8,138 (35.9)	1.9	−2.9†
Sepsis	038	A40–A41	861	2,955 (13.0)	1.4	1.5†
Cardiac	390–398, 420–422	I00–I09, I30–I33, I40	2,702	2,213 (9.8)	0.9	−2.5†
AIDS and HIV	279.5, 279.6, 795.8	B20–B24, R75	NA	944 (4.2)	3.9	NA
Renal/urinary	590, 595, 599.0	N10–N12, N13.6, N15.1, N30, N39.0	474	3,565 (15.7)	1.1	3.1†
Tuberculosis and sequelae	010–018, 137	A15–A19, B90	1,475	284 (1.3)	3.0	−7.0†
Acute respiratory	460–466, 475, 510, 513, 034.0	J00–J08, J20–J22, J36, J85, J86	913	1,136 (5.0)	1.2	−1.6†
Influenza	487	J09–J11	997	214 (0.9)	1.2	−8.0†
Viral hepatitis	070	B15–B19	109	865 (3.8)	1.5	5.7†
Intestinal	001–009	A00–A09	402	719 (3.2)	1.0	−0.6
Other codes						
Infectious and parasitic diseases	020–033, 034.1, 035–037, 039–057, 060–066, 071–136, 138, 139	A20–A39, A42–A99, B00–B14, B25–B89, B91–B99	1,088	586 (2.6)	1.6	−2.9†
Other infectious diseases	320–323, 540–542, 566, 567.0–2, 569.5, 576.1, 770.0, 771	G00–G04, K35–K37, K61.0–4, K63.0, K65.0, K65.8, K67, K83.0, P23, P35–P39	793	1,027 (4.5)	1.4	−0.3

*ICD-9, International Classification of Diseases, 9th revision; ICD-10, International Classification of Diseases, 10th revision; SR, sex ratio (M:F, age-adjusted rate); APC, annual percentage change (age-adjusted rate); NA, not applicable. †Significant because APC CIs did not include 0.

We calculated the crude mortality rates by using population data drawn from NSI population projections. Age- and sex-adjusted rates were calculated by using the direct method and the standard European population as a reference.

We analyzed the trends according to sex, age group (<1–4, 5–24, 25–44, 45–64, and ≥65 years), and for each of the 10 first-selected diseases. We computed the sex ratio of the adjusted rates to assess sex-related differences. Subsequently, we analyzed the trends of death rates by using a joinpoint regression model to estimate the annual percentage change (APC) and to identify trend inflection points (joinpoints) when present. An inflection point was defined as the year representing the final endpoint of 1 period and the initial endpoint of a subsequent period; thus, all periods overlap. For each period, an APC was calculated.

We computed APC for each trend by using generalized linear models and assuming a Poisson distribution (*12*). This analysis initially assumes that there are no joinpoints and iteratively fits models until a curve with the minimum number of joinpoints is selected by using permutation tests (*13*). Adjusted rates and SEs were used to fit all joinpoint models, except for analyses by age group, for which deaths and populations under a Poisson model were used. This method identifies through simulations the minimum number of inflection points (i.e., years when the trend is changing) and quantifies changes in trends through the APC. When the APC is positive and significant, it indicates that the trend is increasing. When the APC is negative and significant, it indicates that the trend is decreasing.

Statistical significance was set at p<0.05. Data analyses were performed by using Stata 12 (StataCorp. LP, College Station, TX, USA) and Joinpoint Regression version 3.5.1 software (http://surveillance.cancer.gov/joinpoint/Joinpoint_Help_4.0.4.pdf). This study was conducted as an activity of infectious disease surveillance at the National Center of Epidemiology, Madrid, Spain.

## Results

During 1980–2011, there were 628,673 deaths caused by infectious diseases in Spain. Although the crude mortality rate decreased from 50.9 to 49.2 deaths/100,000 persons during this period, the adjusted mortality rate decreased from 53.8 to 27.3 deaths/100,000 persons. The joinpoint method identified 2 inflection points in the trend, the first in 1987 and the second in 1994. Adjusted mortality rates indicated a decrease during 1980–1987 and an APC of −6.2% (95% CI –8.5% to –3.9%), an increase during 1987–1994 and an APC of 3.1% (95% CI –0.1% to 6.4%); and a decrease during 1994–2011 and an APC of −2.5% (95% CI –3.1% to –1.9%).

Among men, 4 periods of change were observed. The first period (1980–1987) had an APC of −5.9% (95% CI –8.1% to –3.6%), the second period (1987–1995) had an APC of a 4.9% (95% CI 2.4%–7.2%), the third period (1995–1998) had an APC of −7.8% (95% CI –20.6% to –7.6%), and fourth period (1998–2011) had an APC of −2.5% (95% CI –3.4% to –1.7%). Changes in first, second, and fourth periods were significant because the APC CIs did not include 0.

**Figure 1:**
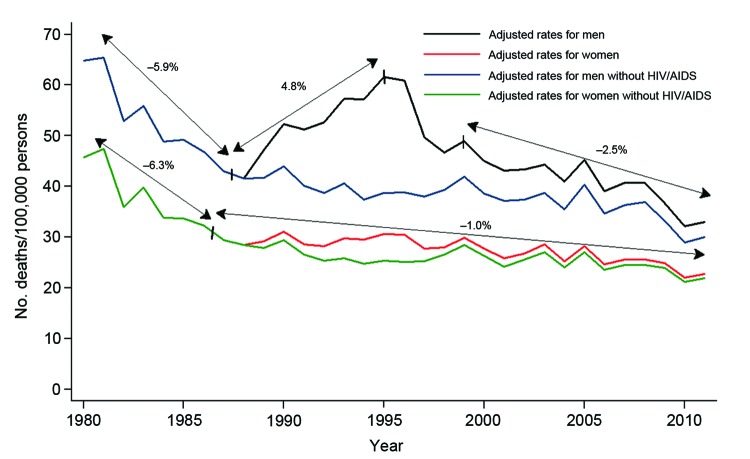
Infectious disease mortality rates by sex, Spain, 1980–2011.

Among women, only 2 periods (1980–1986 and 1986–2011) of change were observed. Both of these changes were significant and showed decreases (APC −6.3%, 95% CI –9.0% to –3.4% and APC −1.0%, 95% CI –1.4% to –0.7%); the inflection point was in 1986 (Figure 1). During 2007–2011, the male:female sex ratio was 1.5:1 for deaths caused by infectious diseases and was higher for 9 of the 10 diseases first selected, except for cardiac infections (Table 1).

**Figure 2:**
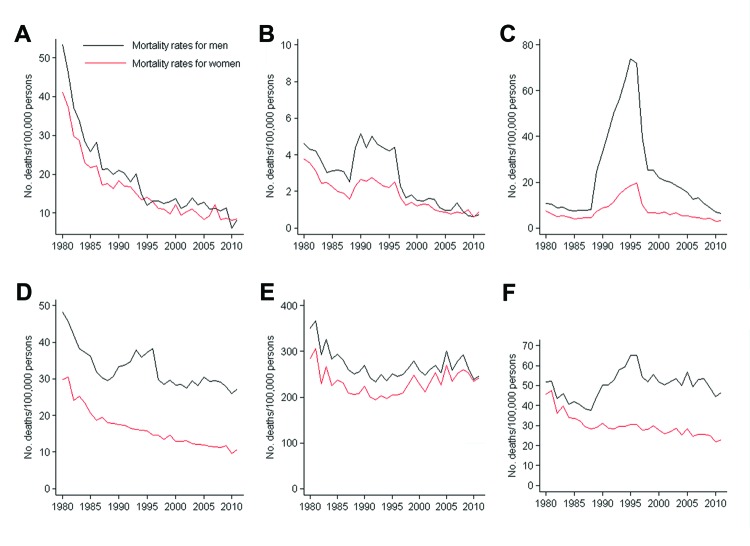
Infectious disease mortality rates by sex and age group, Spain, 1980–2011. A) <1–4 y, B) 5–24 y, C) 25–44 y, D) 45–64 y, E) ≥65 y, F) all ages.

There were major variations in mortality rates by age group and sex; children <1–4 years of age and persons ≥65 years of age showed the largest shifts. Sex-specific mortality rates were higher for male patients across all age groups. Although the largest decrease in deaths caused by infectious disease was among children <1–4 years of age (47.4 deaths/100,000 persons in 1980 and 8.4 deaths/100,000 persons in 2011), the lowest rates were observed among persons 5–24 years of age. A notable peak in deaths occurred in 1989–1997 because of AIDS; the population segment most affected was men 25–44 years of age (Figure 2). The study period showed a major decrease in mortality rates among male and female patients across all age groups. The sharpest decrease was among persons <1–4 years of age, who had an APC of −5.5% (95% CI –6.2% to –4.8%) for male patients and −5.0% (95% CI –5.6% to –4.4%) for female patients. However, among persons >65 years of age, this decrease was much smaller (−0.5%, 95% CI –0.8% to –0.1% for men and −0.1%, 95% CI –0.3% to 0.6% for women) (Table 2).

**Table 2:** Mortality rates for infectious diseases analyzed, by sex, age group, and detected trends, Spain, 1980–2011*

Sex/age group, y	Rate for 1980	Rate for 2011	APC for 1980–2011, %	Trend 1			Trend 2			Trend 3			Trend 4	
				Period	APC, %		Period	APC, %		Period	APC, %		Period	APC, %
M														
<1–4	53.3	8.3	−5.5†	1980–1985	−14.2†		1985–2011	−4.0†		–	–		–	–
5–24	4.6	0.7	−4.2†	1980–1986	−7.7†		1986–1992	10.7†		1992–2011	−11.15†			
25–44	10.7	6.4	−2.8	1980–1985	−4.8		1985–1995	27.0†		1995–1998	−31.3†		1998–2011	−9.7†
45–64	48.1	27.2	−1.4†	1980–1987	−6.7†		1987–1995	3.3†		1995–1998	−8.8		1998–2011	–0.3
≥65	350.2	247.1	−0.5†	1980–1991	−3.3†		1991–2008	1.0†		2008–2011	−5.4		–	–
AR	64.9	33.0	−1.5†	1980–1987	−5.9†		1987–1995	4.8†		1995–1998	−7.7		1998–2011	−2.5†
F														
<1–4	41.1	8.5	−5.0†	1980–1987	−10.9†		1987–2011	−3.3†						
5–24	3.8	0.9	−4.4†	1980–1988	−9.4†		1988–1991	19.1		1991–2011	−7.3†		–	–
25–44	7.4	3.1	−1.7	1980–1986	−9.9†		1986–1995	21.3†		1995–1998	−28.2†		1998–2011	−6.3†
45–64	29.9	10.8	−3.2†	1980–1986	−7.6†		1986–2011	−2.5†		–	–		–	–
≥65	284.0	242.5	−0.1	1980–1989	−4.0†		1989–2011	1.2†		–	–		–	–
AR	45.7	22.70	−1.6†	1980–1986	−6.3†		1986–2011	−1.0†		–	–		–	–

*Rates are no. cases/100,000 persons. APC, annual percentage change (age-adjusted or age-specific rate); –, not applicable; AR, age-adjusted rates. †Significant because APC CIs did not include 0.

**Figure 3:**
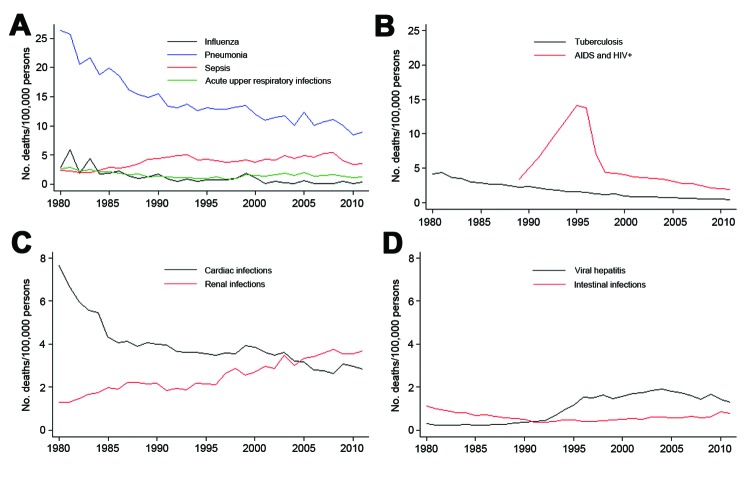
Mortality rates for selected infectious diseases, Spain 1980–2011.

Influenza, pneumonia, acute respiratory infection, and septicemia accounted for 58% of the infectious diseases studied. The mortality rate for pneumonia showed the largest decrease. Starting in 1997, deaths caused by tuberculosis and HIV/AIDS decreased across the study period. Deaths caused by cardiac and renal infections showed mutually opposite trends: deaths caused by cardiac infections decreased gradually over time, and deaths caused by renal infections increased over time. Viral hepatitis and intestinal infections had the lowest mortality rates (Figure 3).

## Discussion

Our results showed that mortality rates for infectious diseases in Spain decreased overall, and that these decreases were only temporarily interrupted by the HIV/AIDS epidemic. Sex-specific mortality rates were higher for male patients across all age groups. Of the 10 first studied diseases, pneumonia showed the largest decrease in mortality rate.

Previous studies showed that analysis of infectious disease mortality rates should take into account infectious disease–related ICD codes from groups other than infectious and parasitic diseases in different ICD revisions because relying only on infectious and parasitic diseases will lead to underestimation of infectious disease mortality rates (*1**,**6**,**10**,**14**,**15*). Using this approach, we showed that 3 times more deaths were caused by infectious disease in Spain in 2011 than when only codes for infectious and parasitic diseases were analyzed, which resulted in a reported underestimated rate (*16*). Our estimated rate was 27.3 deaths/100,000 persons when adjusted for age, with a 1.5-fold increased mortality rate for male patients compared with female patients. Thus, diseases in other ICD diagnostic groups would account for 6% of the general mortality rate, and infectious diseases would be the fourth most common cause of death, instead of the eleventh most common cause of death, if only codes for infectious and parasitic diseases were considered.

Comparison of mortality rates for Spain with those for other countries in Europe is difficult because no standardized data are available, whether because such data solely take the traditional categories into account or because they refer to different periods. For 2010, Eurostat data (*17*) for the European Union (27 member states) showed an adjusted mortality rate of 8.7 deaths/100,000 persons for infectious and parasitic diseases and a male:female sex ratio of 1.7:1.

Our finding of higher mortality rates for male patients for nearly all infectious diseases analyzed, in particular for HIV/AIDS and tuberculosis, was consistent with higher illness rates for male patients because of biologic or behavioral factors (*15**,**18*). In general, these sex-related differences were similar to those for other countries in Europe (*17*).

The HIV/AIDS epidemic in Spain interrupted the decreasing trend of deaths caused by infectious disease in the late 1980s. The initial increase in the HIV/AIDS epidemic was caused mainly by use of shared needles and other items related to injection drug use (*19*). The efficacy of parenteral transmission caused this infection to spread rapidly, and during 1988–1997, Spain had the highest incidence of AIDS in Europe (*20*); the highest incidence was in 1994 (*21*). In 1996, highly active antiretroviral therapy (*21*) was introduced in Spain; it became widely available and was dispensed free of charge. This new treatment resulted in major changes in infectious disease trends in Spain and in mortality rates for AIDS in specific age groups.

In the age group most affected by HIV (persons 25–64 years of age), the imbalance in infection rates between men and women resulted in different trends in infectious disease mortality rates; these mortality rates for men have not yet returned to pre-AIDS rates. For persons 35–44 years of age, infectious diseases accounted for 8% of all deaths in 2011 and were the most frequent cause of death after suicides. For men 25–34 years of age, the mortality rate for other causes has become a more critical issue than the mortality rate for infectious diseases.

In the youngest and oldest age groups, infectious disease mortality rates showed similar trends for both sexes. Children <1–4 years of age showed the sharpest continuous decrease, although this trend has shown a gradual leveling in the past 20 years, which is consistent with low mortality rates for children already observed (*22**,**23*). However, infectious diseases still account for 12% of all deaths in this age group, a finding consistent with reports from other countries (*24**,**25*).

Persons ≥65 years of age were the only age group for whom infectious disease mortality rates increased after the early 1990s, in contrast to a previously decreasing trend (*10*). In this age group, 5 infectious diseases accounted for 4%–7% of all deaths. This trend was essentially caused by deaths of persons in the oldest age group (>80 years), whose relative effect on this trend has increased because of an increase in life expectancy (82 years in 2011). Currently, persons >65 years of age make up 17% of the population of Spain. Of these persons, 14% are >85 years of age. In a health system that provides universal coverage, hospitalization of this frequently immunodeficient elderly population increases the risk for infection. Therefore, septicemia and renal infections are the main causes of the increase in infectious disease mortality rates, as reported for other countries (*26*). However, the 3 other causes (pneumonia, cardiac infections, and respiratory infections), which along with those mentioned, accounted for 90% of the infectious disease mortality rate in elderly persons, have had no role in this increase.

In addition to the increase in septicemia and renal infections, attention should be given to deaths caused by viral hepatitis, a disease that initially showed low mortality rates. However, these rates have increased 9-fold over the past 20 years. This cause of death, which accounted for 4% of deaths caused by infectious diseases, had the highest APC for the study period, and its increase has been especially evident among persons >45 years of age, in particular, those >65 years of age. The increase in deaths caused by viral hepatitis has partly paralleled the AIDS epidemic because both diseases have similar transmission mechanisms. The incidence of hepatitis C has increased in Europe and that of hepatitis B has generally decreased (*27*), probably because of vaccination.

Among relevant infectious respiratory diseases at the beginning of the study period, only pneumonia retained its role as the leading infectious cause of death. Deaths caused by respiratory tuberculosis were surpassed by deaths caused by septicemia, a disease that accounted for 16.6% of deaths caused by infectious diseases. Tuberculosis mortality rates showed a decreasing trend because of lower incidence and efficacy of treatment. AIDS initially led to a reemergence of tuberculosis but has since tended to mask this reemergence, probably because AIDS has being increasingly listed as the underlying cause of death on death certificates.

This study had 3 principal limitations. First, cause-specific death statistics in Spain include only the primary underlying cause of death (*11*), which omit possible contributions of other infectious causes and result in underestimation of deaths caused by infectious diseases, a finding that could be detected only by using multiple coding (*28*). Second, in 1998 Spain replaced ICD-9 with ICD-10, a decision that might have been led to problems of standardization of disease coding and discontinuity in assessment of trends. Third, we were not able to include factors such as improvements in diagnosis and medical practices.

In conclusion, despite a decrease in deaths caused by infectious diseases that was interrupted by HIV/AIDS, infectious diseases continue to be a major cause of death, which is an indicator of their role in public health. In the twenty-first century, the incidence of such diseases might be increased by a series of factors (*7**,**29*), ranging from climate change, which alters the ecology of vectors, to globalization, which involves exchange of goods and mobility of persons for leisure, occupational, or survival purposes. Notable among these factors is excessive use of antimicrobial drugs (*30*) among humans and animals. This excessive use is a fundamental cause of increases in bacterial drug resistance and is responsible for 25,000 deaths annually in Europe (*31*). Newly resistant or multidrug-resistant bacterial strains are more lethal, and emerging or reemerging diseases caused by these strains would be more difficult to control.

In addition to the aforementioned factors, the economic crisis in Europe has resulted in a decrease in health expenditures; public health services have been the most affected by budget cuts (*32*). Among these services, prevention programs, such as disease surveillance and control systems that target populations at risk for infectious diseases, have been greatly affected by underfunding (*32*). In this situation, information provided by vital statistics complements these services for retrospective assessment of trends of infectious disease mortality rates in Spain (*33*).

## References

[1] Armstrong GL, Conn LA, Pinner RW. Trends in infectious disease mortality in the United States during the 20th century. JAMA. 1999;281:61–6. 10.1001/jama.281.1.61 9892452

[2] Bi P, Whitby M, Walker S, Parton KA. Trends in mortality rates for infectious and parasitic diseases in Australia: 1907–1997. Intern Med J. 2003;33:152–62. 10.1046/j.1445-5994.2003.00354.x 12680980

[3] Centers for Disease Control and Prevention. Achievements in public health: control of infectious diseases. MMWR Morb Mortal Wkly Rep. 1999;48:621–9 . 10458535

[4] Arkwright PD, David TJ. Past mortality from infectious diseases and current burden of allergic diseases in England and Wales. Epidemiol Infect. 2005;133:979–84. 10.1017/S0950268805004516 16274494 PMC2870331

[5] Communicable diseases. Health status of Spaniards [in Spanish]. Madrid: Ministerio de Sanidad y Consumo; 1998.

[6] Martínez de Aragón MV, Llácer A, Martínez Navarro JF. Infectious disease mortality in Spain: 1980–1995 [in Spanish]. Boletin Epidemiológico Semanal. 1998;6:165–72.

[7] Jones KE, Patel NG, Levy MA, Storeygard A, Balk D, Gittleman JL, Global trends in emerging infectious diseases. Nature. 2008;451:990–3. 10.1038/nature06536 18288193 PMC5960580

[8] MacLehose L, Mckee M, Weinberg J. Responding to the challenge of communicable disease in Europe. Science. 2002;295:2047–50. 10.1126/science.1070025 11896269

[9] Pinner RW, Teutsch SM, Simonsen L, Klug LA, Graber JM, Clarke MJ, Trends in infectious diseases mortality in the United States. JAMA. 1996;275:189–93. 10.1001/jama.1996.03530270029027 8604170

[10] Fernández de la Hoz K, de Mateo S, Regidor E. Trends in infectious diseases mortality in Spain [in Spanish]. Gac Sanit. 1999;13:256–62 . 10490663 10.1016/s0213-9111(99)71366-x

[11] National Statistics Institute. Methodology of death statistics according to cause of death [cited 2013 Jun 29]. http://www.ine.es/daco/daco42/sanitarias/notaecm.htm

[12] Kim HJ, Fay MP, Feuer EJ, Midthune DN. Permutation tests for joinpoint regression with applications to cancer rates. Stat Med. 2000;19:335–51. 10.1002/(SICI)1097-0258(20000215)19:3<335::AID-SIM336>3.0.CO;2-Z 10649300

[13] Clegg LX, Hankey BF, Tiwari R, Feuer EJ, Edwards BK. Estimating average annual per cent change in trend analysis. Stat Med. 2009;28:3670–82. 10.1002/sim.3733 19856324 PMC2843083

[14] Serraino D, Bidoli E, Piselli P, Angeletti C, Bruzzone S, Pappagallo M, Time trends in infectious disease mortality in Italy: 1969–1999 [in Italian]. Epidemiol Prev. 2004;28:322–9 . 15792154

[15] Shohat T, Harari G, Green MS. Mortality from infectious diseases in Israel, 1979–1992, based on revised ICD-9 codes: implications for international comparisons. Am J Public Health. 1999;89:1855–7. 10.2105/AJPH.89.12.1855 10589316 PMC1509020

[16] National Statistics Institute. Mortality and cause of deaths, 2011 [in Spanish] [cited 2013 Feb 27]. http://www.ine.es/prensa/np767.pdf

[17] European Commision. Populations and social conditions [cited 2013 Jun 30]. http://appsso.eurostat.ec.europa.eu/nui/show.do?dataset=hlth_cd_asdr&lang=en

[18] Owens IP. Ecology and evolution. Sex differences in mortality rate. Science. 2002;297:2008–9. 10.1126/science.1076813 12242430

[19] Díez M, Oliva J, Sanchez F, Vives N, Cevallos C, Izquierdo A. Incidence of new HIV diagnoses in Spain, 2004–2009 [in Spanish]. Gac Sanit. 2012;26:107–15 . 22112715 10.1016/j.gaceta.2011.07.023

[20] European Centre for the Epidemiological Monitoring of AIDS. HIV/AIDS surveillance in Europe. Mid-year report, no. 63. St. Maurice (France): The Center; 2001 [cited 2014 Feb 12]. www.ceses.org/AidsSurv/rapport_n63_2000/rapport63eng.htm

[21] Secretary of the National AIDS Strategy. HIV/AIDS in Spain. Epidemiological situation in 2001 [in Spanish]. Madrid: Ministry of Health; 2002.

[22] Llácer A, Fernández-Cuenca R, Pérez B. Change in the infant mortality in Spain in the last twenty years [in Spanish]. Boletin Epidemiológico Semanal. 2004;23:257–60.

[23] DiLiberti JH, Jackson CR. Long-term trends in childhood infectious disease mortality rates. Am J Public Health. 1999;89:1883–5. 10.2105/AJPH.89.12.1883 10589325 PMC1509028

[24] Read JS, Troendle JF, Klebanoff MA. Infectious disease mortality among infants in the United States, 1983 through 1987. Am J Public Health. 1997;87:192–8. 10.2105/AJPH.87.2.192 9103096 PMC1380793

[25] Wilson D, Bhopal R. Impact of infection on mortality and hospitalization in the North East of England. J Public Health Med. 1998;20:386–95. 10.1093/oxfordjournals.pubmed.a024792 9923944

[26] Angeletti C, Piselli P, Bidoli E, Bruzzone S, Puro V, Girardi E, Analysis of infectious disease mortality in Italy [in Italian]. Infez Med. 2004;12:174–80 . 15711130

[27] Rantala M, van de Laar MJ. Surveillance and epidemiology of hepatitis B and C in Europe - a review. Euro Surveill. 2008;13:1880 . 18761967 10.2807/ese.13.21.18880-en

[28] García Benavides F, Godoy C, Perez S, Bolumar F. Multiple codification of the causes of death: from dying “of” to dying “from” [in Spanish]. Gac Sanit. 1992;6:53–7. 10.1016/S0213-9111(92)71092-9 1624230

[29] Fears R, ter Meulen V. Health benefits of policies to mitigate climate change. Lancet. 2011;377:995–6. 10.1016/S0140-6736(11)60385-1 21420552

[30] Carlet J, Collignon P, Goldmann D, Goossens H, Gyssens IC, Harbarth S, Society's failure to protect a precious resource: antibiotics. Lancet. 2011;378:369–71. 10.1016/S0140-6736(11)60401-7 21477855

[31] European Centre for Disease Prevention and Control. The bacterial challenge: time to react [cited 2013 Jun 30]. http://www.ecdc.europa.eu/en/publications/Publications/0909_TER_The_Bacterial_Challenge_Time_to_React.pdf

[32] Semenza JC, Tsolova S, Lim TA. Economic crisis and infectious disease control: a public health predicament. Eur J Public Health. 2012;22:5–6. 10.1093/eurpub/ckr212 22278723

[33] Rechel B, Suhrcke M, Tsolova S, Suk JE, Desai M, Mckee M, Economic crisis and communicable disease control in Europe: a scoping study among national experts. Health Policy. 2011;103:168–75. 10.1016/j.healthpol.2011.06.013 21820196

